# Supplementary material for: Newly Isolated Virulent Salmophages for Biocontrol of Multidrug-Resistant Salmonella in Ready-to-Eat Plant-Based Food
Source: Int J Mol Sci. 2023 Jun 14;24(12):10134. doi: 10.3390/ijms241210134 (PMC10299301; doi:10.3390/ijms241210134)
Supplement: Supplementary file 1 [file ijms-24-10134-s001.zip › ijms-2371682-Figure S1.pdf]

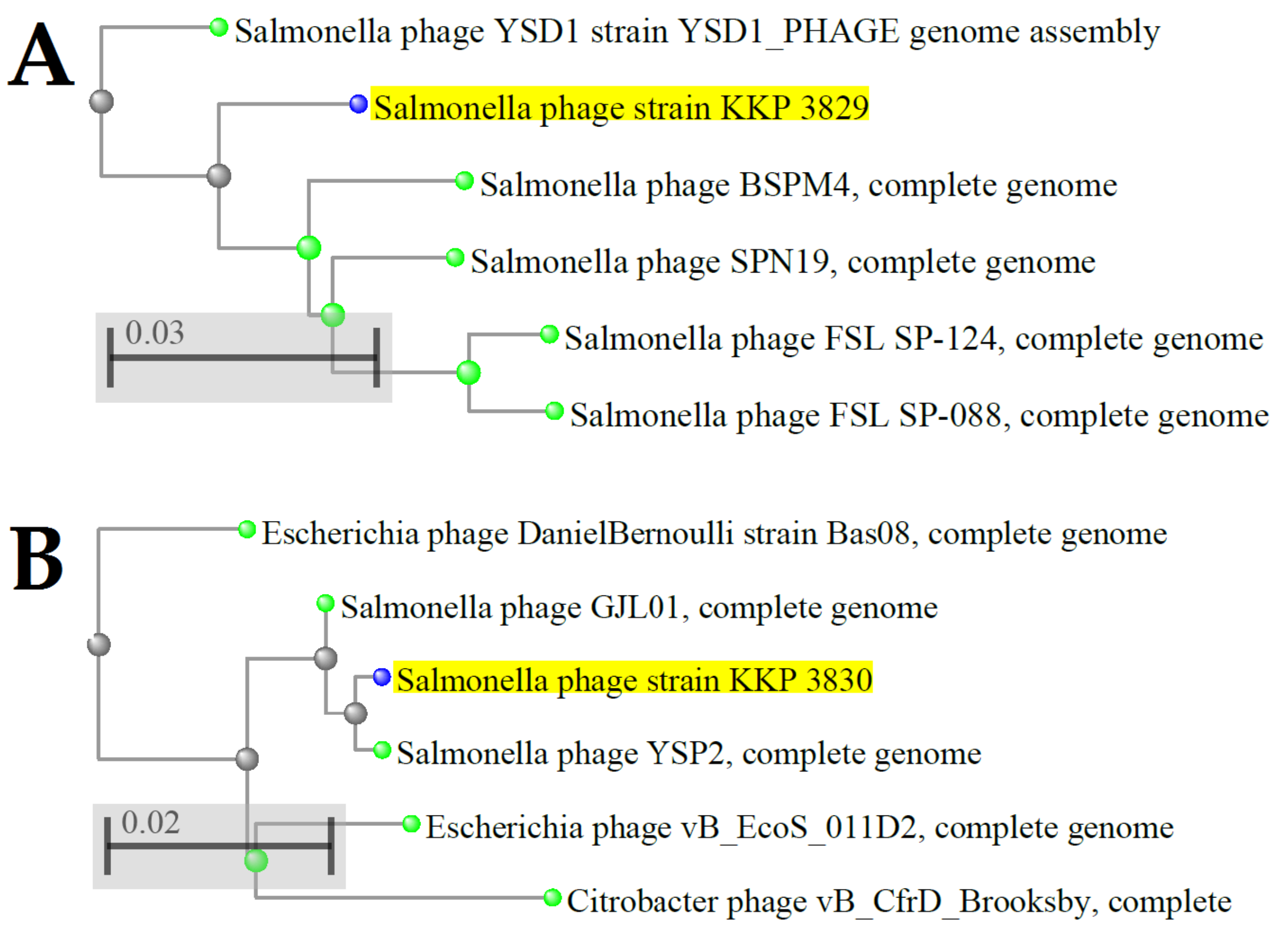

Figure S1. Phylogenetic relatedness of: A – *Salmonella* phage strain KKP 3829; B – *Salmonella* phage strain KKP 3830 against most common phage hits (n = 5) in the NCBI database [97]. The phylogenetic tree was constructed using neighbour-joining method.
