# Supplementary material for: Newly Isolated Virulent Salmophages for Biocontrol of Multidrug-Resistant Salmonella in Ready-to-Eat Plant-Based Food
Source: Int J Mol Sci. 2023 Jun 14;24(12):10134. doi: 10.3390/ijms241210134 (PMC10299301; doi:10.3390/ijms241210134)
Supplement: Supplementary file 1 [file ijms-24-10134-s001.zip › ijms-2371682-Figure S2.pdf]

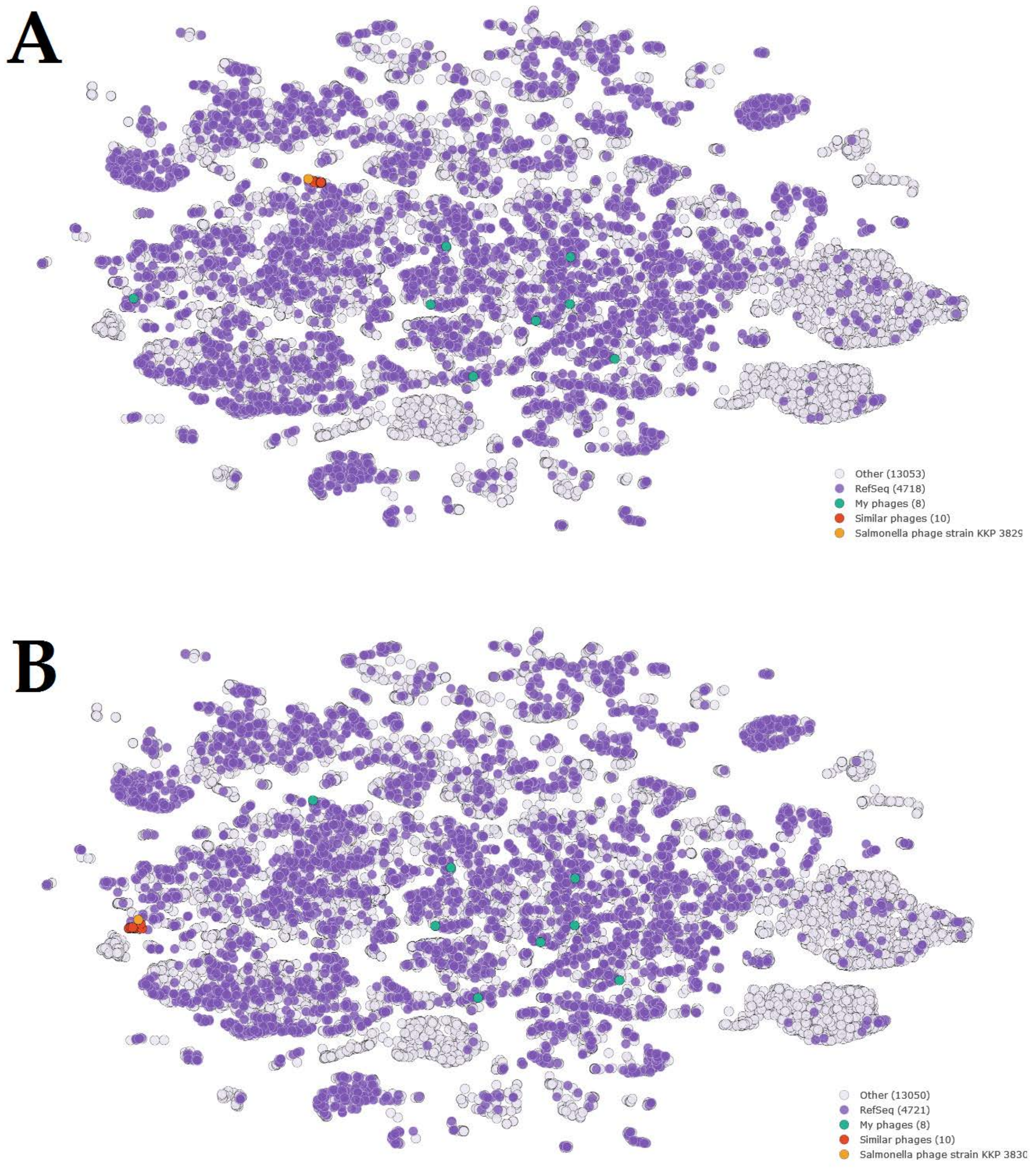

Figure S2. Phage similarity on 2D scatter plot computationally predicted and rendered through PhageAI software [98]: A – *Salmonella* phage strain KKP 3829; B – *Salmonella* phage strain KKP 3830.
