# Supplementary material for: Newly Isolated Virulent Salmophages for Biocontrol of Multidrug-Resistant Salmonella in Ready-to-Eat Plant-Based Food
Source: Int J Mol Sci. 2023 Jun 14;24(12):10134. doi: 10.3390/ijms241210134 (PMC10299301; doi:10.3390/ijms241210134)
Supplement: Supplementary file 1 [file ijms-24-10134-s001.zip › ijms-2371682-Table S2.pdf]

Table S2. BLASTn alignment of homologs phages sequences (at the GenBank database) with isolated salmophages.

Phage (complete genome)	Genome Length [bp]	G+C Content [%]	No. of Genes / Protein	E-Value	Query Coverage [%]	Percent Identity [%]	Phage Family	Genome	Isolated Country	Isolation Source	Lab Host	GenBank Accession No.
***Salmonella* phage strain KKP 3829**												
Salmonella phage FSL SP-124	59,245	56.5	71/71	0.0	96	96.13	*Casjensviridae*	Linear DNA	USA	ND	*Salmonella enterica*	KC139515.1
Salmonella phage FSL SP-088	59,454	56.4	70/70	0.0	96	96.06	*Casjensviridae*	Linear DNA	USA	ND	*Salmonella enterica*	NC_021780.1
Salmonella phage SPN19	59,203	56.5	72/72	0.0	95	96.21	*Casjensviridae*	Circular DNA	South Korea	ND	*Salmonella* sp.	JN871591.1
Salmonella phage YSD1 strain YSD1_PHAGE	58,916	56.5	71/71	0.0	87	96.28	*Casjensviridae*	Circular DNA	United Kingdom	ND	*S.* Typhi	NC_048666.1
Salmonella phage BSPM4	59,097	56.5	78/78	0.0	96	95.69	*Casjensviridae*	Linear DNA	South Korea	swine intestine	*S.* Typhimurium	NC_048655.1
***Salmonella* phage strain KKP 3830**												
Salmonella phage YSP2	50,316	42.9	87/87	0.0	98	99.74	*Drexlerviridae*	Linear DNA	China	sewage	*S.* Pullorum	NC_047898.1
Salmonella phage GJL01	50,407	42.8	80/80	0.0	97	99.77	*Drexlerviridae*	Linear DNA	China	sewage	*S.* Pullorum SP1	KY657202.1
Escherichia phage vB_EcoS_011D2	50,633	42.9	89/89	0.0	93	97.97	*Drexlerviridae*	Linear DNA	China	sewage	*Escherichia coli*	MT478992.1
Escherichia phage DanielBernoulli strain Bas08	50,731	43.2	91/91	0.0	86	96.86	*Drexlerviridae*	Linear DNA	Switzerla nd	sewage plant inflow	*Escherichia coli* K-12	MZ501059.1
Citrobacter phage vB_CfrD_Brooksby	50,071	42.8	86/86	0.0	89	96.94	*Drexlerviridae*	Linear DNA	USA	raw sewage	*Citrobacter freundii*	OL539443.1

ND – no data
